# Risk Factors for Infection in Patients Undergoing Osteosynthesis for Tibial Plateau Fracture in a University Hospital

**DOI:** 10.7759/cureus.24587

**Published:** 2022-04-29

**Authors:** José Eduardo Nogueira Forni, Sérgio Eduardo Tardivo Fraga, Wahi Jalikj

**Affiliations:** 1 Orthopedics and Traumatology, Faculdade de Medicina de São José do Rio Preto (FAMERP), São José do Rio Preto, BRA

**Keywords:** surgery, infection, treatment, bone plates, fractures of the tibia

## Abstract

Schatzker types IV to VI tibial plane fractures compromise the two tibial plateaus. Most cases involve joint deviation and require anatomic reduction and rigid fixation. Dual access and prolonged surgical time are factors that exert an influence on the occurrence of infection of the surgical wound and, consequently, the clinical outcome. The reason why these fractures have a greater incidence of infection compared to others remains unclear. The aim of the present study was to investigate risk factors for infection in patients undergoing osteosynthesis for tibial plateau fracture considering demographic, clinical, and operative factors.

A retrospective study was conducted with data on patients with Schatzker types IV, V, and VI tibial plateau fracture submitted to surgical treatment at a tertiary university hospital affiliated with the public healthcare system. The following data were extracted from the patient files: age; type of fracture; mechanisms of trauma; exposure of fracture; use of external fixator prior to osteosynthesis considering the time of fixator use, distance between Schanz screws, and location of the fracture; presence of compartment syndrome; number of surgical accesses; surgical time; number of participants in surgery and smoking; and comparing groups with and without infection at the surgical site in the immediate postoperative period (up to three weeks). Among the 44 patients studied, mean age was 48.5±15.1 years, 72.7% patients were male, 11.4% were diabetic, 56.8% had Schatzker type V tibial fracture, 88.6% had fractures caused by high-impact trauma, 95.5% of the fractures were closed, 100% used an external fixator prior to definitive osteosynthesis, 54.5% had a single lateral surgical access, and infection at the surgical site occurred in 25% of patients. In the comparison of patients with and without infection, a significant difference was found regarding the distance between the Schanz screws and location of the fracture (p=0.0286), which was shorter in patients with infection at the surgical site. The analysis of potential risk factors for infection revealed that open fracture was the only risk factor in patients with proximal tibial fracture, with a 1.22-fold increase in the likelihood of infection (odds ratios {OR}: 1.22; 95% confidence intervals {CI}: 0.93-1.62; p=0.012). In conclusion, open fracture, greater proximity between the Schanz screws of the external fixator, and the location of the fracture were considered risk factors for infection at the surgical site in patients undergoing osteosynthesis for tibial plateau fracture.

## Introduction

Tibial plateau fracture with joint involvement corresponds to 1.6% of all fractures in adults. Schatzker types IV, V, and VI are common in cases of high-impact trauma, are generally associated with soft tissue injuries, and are difficult to treat, with a high incidence of complications and infection occurring in 9.9% of operated cases [1-3]. The reason why these fractures have a greater incidence of infection compared to other types remains unclear.

Risk factors for infection following osteosynthesis for tibial plateau fracture include open fractures that could not be prevented by surgeons and compartment syndrome, which is directly related to the infection rate of the surgical site due to the need for previous fasciotomy [2], with the rate ranging from 11.8% when the fasciotomy is closed prior to osteosynthesis to 50% when osteosynthesis is performed with open fasciotomy [4,5]. The duration of the surgical procedure is also relevant, as each additional hour of surgery increases the risk of infection at the surgical site in the postoperative period by approximately 78% [6,7]. The use of an external fixator prior to definitive osteosynthesis may be another factor that increases the surgical site infection rate, but it remains unclear whether this increase occurs due to the presence of screws close to the location of the fracture or because fractures that require an external fixator are more severe and have a reserved prognosis [2]. The number of incisions and quantity of osteosynthesis material are also related to an increase in cases of infection due to the increase in surgical time and the handling of soft tissues [7].

On average, surgical site infection increases hospital stay by two weeks and consequently increases the cost of treatment by as much as 300% [8]. According to Metsemakers et al., surgical site infection increases the cost of treatment up to six times, mainly due to the increase in hospital stay and the use of antimicrobials [9]. Besides requiring other surgical interventions, infection contributes to an unsatisfactory outcome and could lead to arthrodesis and amputation [10,11].

The present study is justified by the importance of the analysis of risk factors for infection in patients submitted to osteosynthesis for tibial plateau fracture at a university hospital, as this type of fracture is common in cases of high-impact trauma, is generally associated with soft tissue injuries and has a high occurrence of complications, such as infection, leading to an unsatisfactory clinical outcome and an increase in hospital costs. Moreover, the identification of possible risk factors could contribute to a revision of medical conduct and the establishment of a protocol to enable a reduction in hospital stay and an improvement in the quality of life of these patients. Therefore, the aim of the present study was to investigate risk factors for infection in patients submitted to osteosynthesis for tibial plateau fracture considering demographic, clinical, and operative factors.

## Materials and methods

A retrospective study was conducted with data on patients with type IV, V, and VI tibial plateau fracture submitted to surgical treatment at high complexity university hospital affiliated with the public healthcare system. The study period was from January 2017 to December 2019. Patients undergoing surgical treatment due to other types of tibial plateau fracture were excluded. This study received approval from the institutional review board (certificate number: 40492920.4.0000.5415).

The following data were extracted from the patients' electronic records: age; sex; comorbidities (diabetes and obesity); smoking; time between occurrence of trauma and care; type of trauma; type of fracture; use of external fixator (Valinhos, Brazil: Implantec) prior to osteosynthesis considering time of fixator use, distance between Schanz screws (Valinhos, Brazil: Implantec), and location of the fracture; presence of compartment syndrome; number of surgical accesses; surgical time; number of participants in the surgery; and infection and death. Comparisons were performed between groups with and without the occurrence of surgical site infection in the immediate postoperative period (up to three weeks).

All patients were treated by the same health team in the surgical and hospital setting using osteosynthesis materials from the same company. The same preoperative protocol was used for all patients: (1) patients with Gustilo grade III open fracture were previously treated with a linear external fixator; after the culture results demonstrated no signs of infection and improvement was seen in the surrounding soft parts, these patients were submitted to definitive osteosynthesis; (2) patients with a closed fracture and no considerable damage to soft tissues received definitive osteosynthesis with plates and screws.

Statistical analysis

Descriptive analysis was performed with the calculation of frequencies as well as measures of central tendency and dispersion. The Kolmogorov-Smirnov test was used to determine the normality of quantitative variables. Comparisons between groups with and without infection were then conducted with either the Student’s t-test (parametric data) or Mann-Whitney test (non-parametric data). Comparisons between groups for qualitative variables were performed using Pearson’s chi-squared test.

The calculation of odds ratios (OR) and respective 95% confidence intervals (CI) for the determination of probable risk factors for infection was performed with Pearson’s chi-squared test for qualitative variables. Non-binary qualitative variables were converted into binary variables using the weight of each response option in relation to the risk factor. Quantitative variables were converted into binary qualitative variables based on means or medians (depending on the normality of the data) as the value for classification and always considering the importance of each variable as a risk factor for the attribution of the values 0 (without the factor) and 1 (with the factor). For all analyses, a p-value ≤ 0.05 was considered indicative of statistical significance. The computation programs used were SPSS version 23 (Chicago, IL: SPSS Inc.) and PRISMA version 6.10 (San Diego, CA: GraphPad Software Inc.).

## Results

Among a total of 280 patients with tibial fractures treated in the period analyzed, 44 (15.7%) had Schatzker types IV, V, and VI tibial plateau fractures. The sample was composed of patients ranging in age from 21 to 77 years (mean: 48.5±15.1 years), the male sex (72.7%), diabetics (11.4%), Schatzker type V (56.8%), high-impact trauma (88.6%), closed fracture (95.5%), mean time of 0.5±1.4 days between the occurrence of the trauma and treatment, use of an external fixator prior to definitive osteosynthesis (100%) for a mean period of 10.9±6.2 days, mean distance of 124.5±48.4 mm between the Schanz screws and location of the fracture, single lateral surgical access (54.5%), mean surgical time of 189.8±51.1 minutes, four participants during the surgical procedure (47.7%), and infection of the surgical site (25%) (Tables 1, 2).

**Table 1:** Characterization of patients with proximal tibial fracture submitted to surgical treatment at a university hospital from 2017 to 2019 (n=44).

Variables		n (%)
Sex	Female	12 (27.3)
	Male	32 (72.7)
Comorbidities	Diabetes	5 (11.4)
	Smoking	3 (6.8)
	Obesity	2 (4.5)
Schatzker classification	IV	5 (11.4)
	V	25 (56.8)
	VI	14 (31.8)
Trauma	Low impact	5 (11.4)
	High impact	39 (88.6)
Fracture	Closed	42 (95.5)
	Open	2 (4.5)
	Prior use of external fixator	44 (100)
Compartment syndrome	Presence	0 (0.0)
	Absence	44 (100.0)
Surgical access	Single - lateral	24 (54.5)
	Dual - medial and lateral	20 (45.5)
Number of participants in surgery	2	3 (6.8)
	3	16 (36.4)
	4	21 (47.7)
	5	4 (9.1)
Infection at surgical site	Presence	11 (25)
	Absence	33 (75)
	Death	0 (00)

**Table 2:** Descriptive statistics of patients with proximal tibial fracture submitted to surgical treatment at a university hospital from 2017 to 2019 (n=44).

Variable	Mean	SD	Median	Minimum	Maximum
Age (years)	48.5	15.1	49.0	21.0	77.0
Time between trauma and care (days)	0.5	1.4	0.0	0.0	7.0
Duration of use of external fixator (days)	10.9	6.2	10.0	3.0	30.0
Distance between screws and fracture (mm)	124.5	48.4	132.0	0.0	230.0
Duration of surgery (minutes)	189.8	51.1	187.5	108.0	335.0

In the comparison of patients with and without infection, a significant difference was found regarding the distance between the Schanz screws and location of the fracture (p=0.0286), which was shorter in the patients with infection at the surgical site (Table 3). No significant differences between groups were found regarding age, time between trauma and care, duration of external fixator use, duration of surgery, or number of participants in the surgical procedure.

**Table 3:** Mean and standard deviation of variables of patients with proximal tibial fracture submitted to surgical treatment at a university hospital with (n=11) and without (n=33) infection from 2017 to 2019. *Significant difference.

Variable	Infection	No infection	p-Value
Age (years)	51.1±16.0	47.6±15.0	0.5142
Time between trauma and care (days)	0.2±0.6	0.6±1.5	0.2513
Duration of use of external fixator (days)	11.0±4.7	10.9±6.7	0.5678
Distance between screws and fracture (mm)	97.1±47.3	133.6±45.9	0.0286*
Duration of surgery (minutes)	193.0±71.0	188.8±43.9	0.8146
Number of participants in surgery	3.5±0.9	3.6±0.7	0.6366

The analysis of the distribution of potential risk factors for infection with the calculation of ORs and respective 95% confidence intervals (CI) using Pearson’s chi-squared test revealed that open fracture was the only variable considered a risk factor for infection in patients with proximal tibial fracture, with a 1.22-fold greater likelihood of infection in these patients (OR: 1.22; 95% CI: 0.93-1.62; p=0.012) (Table 4).

**Table 4:** Distribution of potential risk factors for infection, odds ratio (OR), 95% confidence interval (CI) in patients with proximal tibial fracture submitted to surgical treatment at a university hospital from 2017 to 2019 (n=44). *Significant difference.

Variable		Infection, n=11 (%)	No infection, n=33 (%)	OR (95% CI)	p-Value
Age	<49 years	5 (45.5)	17 (51.5)	1.28 (0.32-5.01)	0.728
	>49 years	6 (54.5)	16 (48.5)	-	-
Smoking	Absence	11 (100.0)	30 (90.9)	0.91 (0.82-1.01)	0.300
	Presence	0 (0.0)	3 (9.1)	-	-
Diabetes	Absence	9 (81.8)	30 (90.9)	2.22 (0.32-15.43)	0.411
	Presence	2 (18.2)	3 (9.1)	-	-
Obesity	Absence	10 (90.9)	32 (97.0)	3.20 (0.18-55.95)	0.403
	Presence	1 (9.1)	1 (3.0)	-	-
Trauma	Low impact	1 (9.1)	4 (12.1)	1.38 (0.14-13.84)	0.784
	High impact	10 (90.9)	29 (87.9)	-	-
Time between trauma/care	<0 days	10 (90.9)	24 (72.7)	0.27 (0.03-2.39)	0.213
	>0 days	1 (9.1)	9 (27.3)	-	-
Open fracture	Absence	9 (81.8)	33 (100.0)	1.22 (0.93-1.62)	0.012*
	Presence	2 (18.2)	0 (0.00)	-	-
Duration of fixator use	<10 days	4 (36.4)	17 (51.5)	1.86 (0.46-7.58)	0.384
	>10 days	7 (63.6)	16 (48.5)	-	-
Surgical access	Single - lateral	6 (54.5)	18 (54.5)	1.00 (0.25-3.94)	1.000
	Dual - medial and lateral	5 (45.5)	15 (45.5)	-	-
Duration of surgery	<187.5 minutes	6 (54.5)	16 (48.5)	0.78 (0.20-3.08)	0.728
	>187.5 minutes	5 (45.5)	17 (51.5)	-	-
Participants in surgery	<4	10 (90.9)	30 (90.9)	1.11 (0.01-1.19)	0.071
	>4	1 (9.1)	3 (9.1)	-	-
Distances Schanz screws/fracture	<132 mm	3 (27.3)	19 (57.6)	3.62 (0.81-16.16)	0.162
	>132 mm	8 (72.7)	14 (42.4)	-	-

## Discussion

The present study showed that open fracture, the distance between the Schanz screws, and location of the fracture were considered risk factors for surgical site infection in the immediate postoperative period in patients with Schatzker types IV, V, and VI tibial plateau fracture submitted to surgical treatment at a high complexity university hospital affiliated with the public healthcare system.

Surgical site infection occurs in 9.9% of cases of osteosynthesis for tibial plateau fracture [2] and is a determinant of an unsatisfactory outcome due to the need for further surgical interventions to identify the infectious agent and perform the mechanical cleansing of the fracture, which lengthens the hospital stay and increases treatment costs by as much as 300% [8,12]. The main factors that favor infection in this type of osteosynthesis are open fracture, fracture type (Schatzker V/VI), compartment syndrome, smoking, diabetes, and the presence of an external fixator prior to surgery [2,3,13]. In the present series of 44 patients with tibial plateau fracture operated by the same surgical team, acute surgical site infection occurred in 11 (25%) patients.

The distance between the Schanz screws and location of the fracture was identified as a risk factor for infection in the present study. The average distance in patients with infection (97.1 mm) was significantly shorter compared to those without infection (133.6 mm), showing greater proximity of the screws to the fracture. The presence of Schanz screws communicating with the external environment and the proximity of the screws to the surgical access site may explain the increased risk of infection. This finding suggests the need to alter the surgical conduct, with the positioning of the Schanz screws as far as possible from the fracture, which may reduce the occurrence of acute infection of the osteosynthesis.

In the analysis of potential risk factors for infection in patients with tibial plateau fracture, open fracture was the only variable that significantly increased the likelihood of infection in the immediate postoperative period (OR: 1.22; 95% CI: 0.93-1.62; p=0.012). This finding is in agreement with data reported in the literature [2,7,13,14]. It should be pointed out that, while open fracture contributes to the occurrence of infection, it cannot be controlled by the medical team.

No significant differences were found between patients with and without infection regarding age, time between trauma and care, duration of external fixator, duration of surgery, or number of participants involved in the surgical procedure. This differs from findings reported in previous studies regarding the duration of fixator use and duration of surgery [2,7,13]. According to the literature, each additional hour of surgery increases the risk of surgical site infection in the postoperative period by approximately 78% [6,7]. No significant differences between groups undergoing osteosynthesis for tibial plateau fracture with and without infection were found with regards to obesity, diabetes, or smoking. In contrast, some researchers found that smoking was a risk factor for surgical site infection in the same type of fracture [2,14,15].

The need for two surgical access sites with considerable dissection of soft tissues and a prolonged surgical time favor the occurrence of complications, such as deep vein thrombosis, compartment syndrome, and surgical site infection [7]. However, no significant differences between groups with and without infection were found regarding the number of surgical accesses or duration of surgery.

The identification of risk factors for acute infection at the surgical site is essential. Such knowledge can assist in establishing protocols for minimizing or even preventing complications associated with this type of surgery. This information could also be used as an important indicator of the quality of care in the postoperative period provided in the public healthcare setting. The study's limitation is that it used retrospective data from a university hospital and had a small sample size. Therefore, prospective, multi-center studies are needed to confirm the results.

## Conclusions

In the present study, open fracture, greater proximity between the Schanz screws of the external fixator, and the location of the fracture were considered risk factors for infection at the surgical site in the immediate postoperative period in patients with Schatzker types IV, V, and VI tibial plateau fracture undergoing surgical treatment at a high complexity university hospital affiliated with the public healthcare system. The identification of risk factors for acute infection at the surgical site is essential. Such knowledge can assist in establishing protocols for minimizing or even preventing complications associated with this type of surgery.
